# Registration, publication, and outcome reporting among pivotal clinical trials that supported FDA approval of high-risk cardiovascular devices before and after FDAAA

**DOI:** 10.1186/s13063-021-05790-9

**Published:** 2021-11-17

**Authors:** Matthew J. Swanson, James L. Johnston, Joseph S. Ross

**Affiliations:** 1 Frank H. Netter MD School of Medicine at Quinnipiac University, North Haven, CT USA; 2 Yale School of Medicine, New Haven, CT USA; 3 Section of General Medicine and the National Clinician Scholars Program, Department of Internal Medicine, Yale School of Medicine, PO Box 208093, New Haven, CT 06520 USA; 4 Department of Health Policy and Management, Yale School of Public Health, New Haven, CT USA; 5 Center for Outcomes Research and Evaluation, Yale-New Haven Hospital, New Haven, CT USA

**Keywords:** Clinical trials, Publications, Device approval, United States Food and Drug Administration

## Abstract

**Background:**

Selective registration, publication, and outcome reporting of clinical trials distort the primary clinical evidence that is available to patients and clinicians regarding the safety and efficacy of US Food and Drug Administration (FDA)-approved medical devices. The purpose of this study is to compare registration, publication, and outcome reporting among pivotal clinical trials that supported FDA approval of high-risk (class III) cardiovascular devices before and after the FDA Amendment Act (FDAAA) was enacted in 2007.

**Methods:**

Using publicly available data from ClinicalTrials.gov, FDA summaries, and PubMed, we determined registration, publication, and reporting of findings for all pivotal clinical studies supporting FDA approval of new high-risk cardiovascular devices between 2005 and 2020, before and after FDAAA. For published studies, we compared both the primary efficacy outcome with the FDA’s Premarket Approval (PMA) primary efficacy outcome and the published interpretation of findings with the FDA reviewer’s interpretation (positive, equivocal, or negative).

**Results:**

Between 2005 and 2020, the FDA approved 156 high-risk cardiovascular devices on the basis of 165 pivotal trials, 48 (29%) of which were categorized as pre-FDAAA and 117 (71%) as post-FDAAA. Post-FDAAA, pivotal clinical trials were more likely to be registered (115 of 117 (98%) vs 24 of 48 (50%); *p* < 0.001), to report results (98 of 117 (87%) vs 7 of 48 (15%); *p* < 0.001) on ClinicalTrials.gov, and to be published (100 or 117 (85%) vs 28 of 48 (58%); *p* < 0.001) in peer-reviewed literature when compared to pre-FDAAA. Among published trials, rates of concordant primary efficacy outcome reporting were not significantly different between pre-FDAAA trials and post-FDAAA trials (24 of 28 (86%) vs 96 of 100 (96%); *p* = 0.07), nor were rates of concordant trial interpretation (27 of 28 (96%) vs 93 of 100 (93%); *p* = 0.44).

**Conclusions:**

FDAAA was associated with increased registration, result reporting, and publication for trials supporting FDA approval of high-risk medical devices. Among published trials, rates of accurate primary efficacy outcome reporting and trial interpretation were high and no different post-FDAAA.

## Background

In 1976, Congress passed the Medical Device Amendments to the Food, Drug, and Cosmetic (FD&C) Act. This act established three classes for medical devices based on the regulatory controls necessary to provide reasonable assurance of their safety and efficacy [1, 2]. The most tightly regulated devices—those that support or sustain human life, are of substantial importance in preventing impairment of human health, or could pose an unreasonable risk of illness or injury—are categorized as class III (high-risk) devices [2]. High-risk medical devices, such as stents, valves, sealants, and catheters, are regulated through the FDA’s Premarket Approval (PMA) pathway. Under the PMA pathway, device manufacturers are required to submit premarket clinical evidence that provides reasonable assurance of device safety and effectiveness [3]. Pivotal clinical studies are generally the primary clinical evidence on which the FDA bases its approval decisions because they are designed to meet the aforementioned regulatory requirements, demonstrating both the safety and efficacy of the device for the intended use [4]. Given the widespread use of high-risk medical devices in clinical practice, the quality and transparency of clinical data supporting their approval are of paramount importance to patient health and well-being [5].

Between January 2000 and December 2010, less than 50% of studies supporting PMA of novel, high-risk cardiovascular devices were published, and more than 30% of these publications presented primary endpoint results that were different, or could not be compared, to those in the corresponding FDA documents [6]. Discrepancies between registered and published outcomes of clinical trials are common [7]. These practices, known as selective publication and selective outcome reporting, distort the evidence available to patients and clinicians when making care decisions regarding the use of medical devices [8–10]. In 2007, the US FDA Amendment Act (FDAAA) was enacted, mandating clinical trial registration and result reporting on ClinicalTrials.gov for all ongoing and forthcoming trials of FDA-regulated products [8, 11, 12]. It has been reported that post-enactment of FDAAA, pivotal efficacy trials supporting the approval of new drugs for cardiovascular disease, diabetes mellitus, and neuropsychiatric disease were significantly more likely to be registered, be published, and have reported outcomes concordant with those submitted to FDA [9, 13, 14]. However, no studies have examined the impact of FDAAA on the registration and reporting of clinical trials supporting FDA approval of medical devices.

Accordingly, we sought to characterize registration, result reporting, publication, and outcome reporting for pivotal studies supporting high-risk cardiovascular devices before and after the implementation of FDAAA. We focused on cardiovascular devices because they account for more than half of all FDA PMAs [15]. Furthermore, we focused on pivotal studies because they are the definitive studies designed to evaluate medical device safety and effectiveness that are used as the basis of FDA’s regulatory decisions [15, 16]. The results of our study will inform future policy and regulatory efforts to ensure transparency and unbiased results reporting of the clinical trials supporting FDA approval of high-risk medical devices.

## Methods

### Identification of high-risk cardiovascular medical devices

**Fig. 1:**
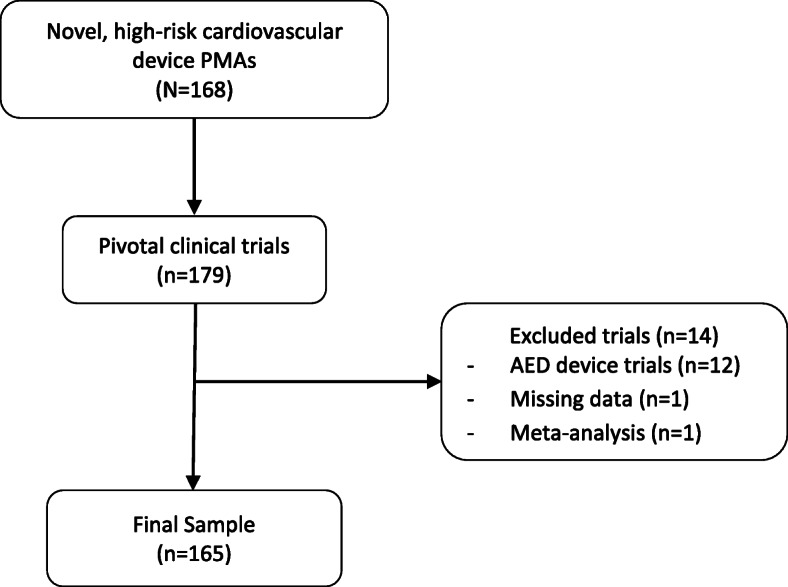
Sample construction of the novel, high-risk cardiovascular devices approved by the US FDA between 2005 and 2020

One author (MJS) identified novel, high-risk cardiovascular medical devices from the publicly accessible FDA PMA database (www.accessdata.fda.gov/scripts/cdrh/cfdocs/cfpma/pma.cfm) between January 1, 2005, and January 1, 2020, excluding automated external defibrillators (AEDs), studies that had missing data, and summaries that leveraged a meta-analysis for the pivotal study (Fig. 1). We excluded AEDs because FDA published a final order on January 29, 2015, stating that AED clinical study information can be leveraged from both published studies and clinical data previously submitted under the 510(k) process instead of requiring the conduct of a pivotal trial to support FDA approval [17]. Otherwise, devices were selected if they met both of the following parameters: “Cardiovascular” under advisory committee and “Originals Only” under supplement type. All devices were characterized by the following using publicly available information on the FDA website: FDA review type (priority/standard), implantable designation (yes/no), life-sustaining designation (yes/no), and combination product (yes/no). Sponsor company management (public/private) was also determined by Google searching the sponsor company name along with “publicly traded,” “stock price,” “IPO,” or “privately held.”

### Characterization of pivotal clinical trials

For each device, one author (MJS) then identified the pivotal clinical studies that supported device approval from the “Summary of Safety and Effectiveness” documents. Pivotal clinical studies supporting approvals were categorized as pre-FDAAA if the clinical trial primary completion date was before December 26, 2007 (the date the policy took effect), in a manner described previously [9]; all other studies were categorized as post-FDAAA. Also, we categorized pivotal trials by specific design characteristics: use of randomization (yes/no), use of blinded allocation (yes/no), primary efficacy endpoint (surrogate marker/clinical outcome or scale), and study center and patient enrollment numbers. These metrics have been widely reported as assessing the validity and quality of clinical trials [9, 13, 18–20].

Study characteristics and data were abstracted from the FDA summaries by one author (MJS); a 10% subsample (17 devices) was randomly selected for validation by a second author (JLJ) at the beginning of data extraction. There were no disagreements in the 7 product/study characteristics extracted from FDA summaries and other sources (e.g., FDA review pathway, life-sustaining designation, implantable designation, combination product, company management, and use of randomization and blinding): percent agreement = 100% (119/119).

Following this independent validation, JLJ verified all device extractions for which MJS had been unable to identify clinical trial registration, result reporting, or publication, or for which MJS determined there was discordance in results reported or trial interpretation. There were a total of 132 transparency measure extractions for these devices, on which MJS and JLJ agreed on 97 and disagreed on 35 (percent agreement of 73.5%). Among these 35 disagreements, 3 related to trial registration, 5 to result reporting, 18 to publication, 8 to result concordance, and 1 to interpretation concordance. All disagreements were resolved via consensus among all authors. This process demonstrated that the initial search was overly reliant on the ClinicalTrials.gov hyperlinks, which were not always accurately reported and do not reliably identify trial publications [21]. Afterwards, the search strategy was revised and repeated to identify publications using and comparing clinical trial titles, product names, methods, number of study centers, enrollment numbers, primary efficacy endpoints, primary results, and study sponsors.

### Identification of trials on ClinicalTrials.gov and published in the peer-reviewed literature

For each pivotal trial identified from FDA documents, we conducted a comprehensive search of ClinicalTrials.gov and PubMed’s listing of MEDLINE-indexed journals to identify any corresponding trial registration or publication, respectively. One author (MJS) conducted the initial search; a second author (JLJ) reviewed all pivotal trials for which a clinical trial registration or publication was not identified after the initial search by MJS; differences (*n* = 3 and 18, respectively) were reconciled by consensus among all authors. All document and website searches were performed during July 2020. Our search strategy included using and comparing clinical trial titles, product names, methods, number of study centers, enrollment numbers, primary efficacy endpoints, primary results, and study sponsors. While more recent FDA PMAs include ClinicalTrials.gov registration hyperlinks and ClinicalTrials.gov manually and automatically indexes corresponding publications of results to their registration by National Clinical Trial (NCT) number, these identification numbers did not reliably identify pivotal trial registrations and publications for older PMAs, consistent with prior reviews [21]. Among publications identified in PubMed, abstracts and conference reports were excluded. Publications reporting multiple studies, such as reviews and meta-analyses, were also excluded unless the results of each study were analyzed and discussed individually at the level of detail as one would expect from a full-length publication.

### Comparison to corresponding publications

First, for each pivotal trial for which a publication was identified, we compared the primary effectiveness endpoint specified in the FDA documents with the effectiveness endpoint specified as primary in the publication. If there was more than one primary effectiveness endpoint reported in the FDA documents, we verified the one that matched the primary endpoint specified in the publication. If none of the specified primary endpoints matched, we categorized the primary effectiveness outcomes reported as discordant. If one matched, we determined whether the primary effectiveness endpoint result reported in the FDA documents was the same as the result reported in the publication. The outcomes reported were categorized as concordant if they shared all five defined elements of an endpoint (i.e., domain, measure, metric, method of aggregation, and timepoint) and were an exact numerical match or if there was a relative difference of less than 5% when compared to the FDA PMA, a conservative estimate intending to identify clinically meaningful differences while recognizing that there might be changes in analytical approaches over time [22]. Otherwise, the outcomes reported were categorized as discordant, as well as if the documented primary endpoint in the FDA materials was included in the publication but reported as a secondary outcome and if the primary endpoint in the FDA documentation pre-specified with the FDA was switched by the sponsor in their FDA documentation from what was pre-specified in a protocol or a registry entry. Second, for each pivotal trial for which a publication in the peer-reviewed literature was identified, we compared the overall study interpretation between the two sources. The overall interpretation was categorized as positive, equivocal, or negative based on the FDA officer’s language in the “Effectiveness Conclusions” and “Overall Conclusions” subsections of the “Summary of Safety and Effectiveness” document and the author’s language in the conclusion of the publication; the FDA and publication interpretation were categorized as concordant or discordant. One author (MJS) conducted the outcome comparisons; a second author (JLJ) verified the outcome comparisons classified as discordant by MJS and differences were reconciled by consensus among all authors.

### Statistical analysis

We determined the rate of ClinicalTrials.gov registration, ClinicalTrials.gov result reporting, and PubMed publication for all identified pivotal trials, overall and stratified by device and design characteristics. We then determined the overall rate of concordant primary outcome reporting between the FDA PMA summaries and corresponding publications, as well as the overall rate of concordance between the FDA PMA reviewer’s interpretations and the trial publication’s interpretations. Summary statistics were calculated for each comparison, presented as numbers, percentages, means, standard deviations, and ranges, as appropriate. Chi-square and two-tailed Fisher exact tests were used to compare rates pre- and post-FDAAA of registration, result reporting, publication, concordant outcome reporting, and concordant interpretation, as appropriate. All statistical tests were two-tailed and used the Bonferroni method to correct our alpha value to account for multiple comparisons of five transparency measures across the sample of clinical trials for these devices: (1) registration, (2) result reporting, (3) publication, (4) concordant outcome reporting, and (5) concordant interpretation. Statistical significance was set at *p* ≤ 0.01. Analyses were performed using Microsoft Excel (version 16.35) and SPSS (version 27).

### Research guidelines and ethics

This study was prepared in accordance with the Strengthening the Reporting of Observational Studies in Epidemiology (STROBE) reporting guideline for cross-sectional studies [23]. The study did not require institutional review board approval or patient informed consent because it was based on publicly available information and involved no patient records.

## Results

Between 2005 and 2020, the FDA approved 156 novel, high-risk cardiovascular devices (Table 1). Among these, 16 (10%) approvals were designated for priority review, 84 (54%) were life-sustaining, 123 (79%) were implantable, 29 (19%) were combination products, and 60 (38%) had private sponsor company management.

**Table 1:** Novel, high-risk cardiovascular devices approved by the US FDA between 2005 and 2020

Novel approvals (*N* = 156)	Number (%)
FDA review pathway^a^	
Priority	16 (10)
Standard	140 (90)
Life-sustaining designation^a^	
Life-sustaining	84 (54)
Non-life-sustaining	72 (46)
Implantable designation^a^	
Implantable	123 (79)
Non-implantable	33 (21)
Combination product^a^	
No	127 (81)
Yes	29 (19)
Company management	
Public	96 (62)
Private	60 (38)

*FDA* US Food and Drug Administration

^a^FDA designation

We identified a total of 179 pivotal clinical trials supporting these 156 approvals, of which 165 met our inclusion criteria (Fig. 1), among which 48 (29%) were categorized as pre-FDAAA and 117 (71%) as post-FDAAA. Overall, 59 (36%) of these pivotal trials were randomized, 21 (13%) were blinded, 103 (62%) were from publicly held companies, 19 (12%) supported devices with priority review status, 32 (19%) supported combination products, 132 (80%) supported implantable devices, and 89 (54%) supported life-sustaining devices.

### Trial registration, result reporting, and publication

Among the 165 pivotal trials that met our inclusion criteria, 139 (84%) were registered on ClinicalTrials.gov, 105 (76%) had results posted on ClinicalTrials.gov, and 128 (78%) were published in the peer-reviewed literature. Compared to pre-FDAAA pivotal trials, post-FDAAA trials were more likely to be registered on ClinicalTrials.gov (115 of 117 (98%) vs 24 of 48 (50%); *p* < 0.001), to report results on ClinicalTrials.gov (98 of 117 (84%) vs 7 of 48 (15%); *p* < 0.001), and to be published (100 of 117 (85%) vs 28 of 48 (58%); *p* < 0.001) (Table 2). Trials registered on ClinicalTrials.gov were more likely to be published than those not registered (121 of 139 (87%) vs 7 of 26 (27%); *p* < 0.001). Pre-FDAAA, implantable designation was associated with a greater likelihood of registration (21 of 36 (58%) vs 3 of 12 (25%); *p* = 0.05) and life-sustaining designation was associated with a greater likelihood of result reporting or publication (18 of 24 (75%) vs 11 of 24 (46%); *p* = 0.04; Table 3). Post-FDAAA, there were no significant differences in registration, result reporting, or publication when stratified by device or trial design characteristics (Table 4).

**Table 2:** Registration, result reporting, and publication of clinical trials supporting US FDA cardiovascular device approvals between 2005 and 2020, pre- and post-FDAAA (*n* = 165)

	Number of studies (%)	Registered on CT.gov, *n* (%)^a^	Risk ratio (*95% CI*)	Report results on CT.gov, *n* (%)	Risk ratio (*95% CI*)	Published, *n* (%)^a^	Risk ratio (*95% CI*)
Overall	165 (100)	139 (84)		105 (76)		128 (78)	
Trial completion date			1.97 (1.48 to 2.61)		5.74 (2.88 to 11.44)		1.47 (1.14 to 1.88)
Pre-FDAAA	48 (29)	24 (50)		7 (15)		28 (58)	
Post-FDAAA	117 (71)	115 (98)		98 (84)		100 (85)	
*p* value			< 0.001		< 0.001		< 0.001

*CT.gov* ClinicalTrials.gov, *CI* confidence interval, *FDAAA* FDA Amendment Act

**Table 3:** Registration and result reporting or publication of clinical trials supporting US FDA cardiovascular device approvals between 2005 and 2020, stratified by study and device characteristics, pre-FDAAA (*n* = 48)

	Number of studies (%)	Registered on CT.gov, *n* (%)	*p* value	Report results on CT.gov or published, *n* (%)	*p* value
Pre-FDAAA	48 (100)	24 (50)		29 (60)	
FDA review pathway^a^			0.76		0.36
Priority	2 (4)	1 (50)		2 (100)	
Standard	46 (96)	23 (50)		27 (59)	
Life-sustaining designation^a^			0.08		0.04
Life-sustaining	24 (50)	15 (63)		18 (75)	
Non-life-sustaining	24 (50)	9 (38)		11 (46)	
Implantable designation^a^			0.05		0.12
Non-implantable	12 (25)	3 (25)		5 (42)	
Implantable	36 (75)	21 (58)		24 (67)	
Combination product^a^			0.12		0.31
No	40 (83)	18 (45)		23 (58)	
Yes	8 (17)	6 (75)		6 (75)	
Company management			0.38		0.37
Private	19 (40)	8 (42)		10 (53)	
Public	29 (60)	16 (55)		19 (66)	
Randomized design			1.00		0.30
No	32 (67)	16 (50)		21 (66)	
Yes	16 (33)	8 (50)		8 (50)	
Blinded design			0.17		0.67
No	43 (90)	20 (47)		26 (60)	
Yes	5 (10)	4 (80)		3 (60)	

*CT.gov* ClinicalTrials.gov, *FDA* US Food and Drug Administration, *FDAAA* FDA Amendment Act

^a^FDA designation

**Table 4:** Registration and result reporting or publication of clinical trials supporting US FDA cardiovascular device approvals between 2005 and 2020, stratified by study and device characteristics, post-FDAAA (*n* = 117)

	Number of studies (%)	Registered on CT.gov, n (%)	*p* value	Report results on CT.gov or published, n (%)	*p* value
Post-FDAAA	117 (100)	115 (98)		114 (97)	
FDA review pathway^a^			0.73		0.62
Priority	17 (15)	17 (100)		17 (100)	
Standard	100 (86)	98 (98)		97 (97)	
Life-sustaining designation^a^			0.20		0.42
Life-sustaining	65 (56)	65 (100)		64 (98)	
Non-life-sustaining	52 (44)	50 (96)		50 (96)	
Implantable designation^a^			0.67		0.55
Non-implantable	21 (18)	21 (100)		21 (100)	
Implantable	96 (82)	94 (98)		93 (97)	
Combination product^a^			0.63		0.50
No	93 (80)	91 (98)		91 (98)	
Yes	24 (21)	24 (100)		23 (96)	
Company management			0.63		0.70
Private	43 (37)	42 (98)		43 (100)	
Public	74 (63)	73 (99)		72 (97)	
Randomized design			0.60		0.25
No	74 (63)	73 (99)		71 (96)	
Yes	43 (37)	42 (98)		43 (100)	
Blinded design			0.26		0.64
No	101 (86)	100 (99)		98 (97)	
Yes	16 (14)	15 (94)		16 (100)	

*CT.gov* ClinicalTrials.gov, *FDA* US Food and Drug Administration, *FDAAA* FDA Amendment Act

^a^FDA designation

### Concordant outcome reporting and trial interpretation

A primary effectiveness outcome and the main result were reported in the FDA documents for all pivotal trials, pre- and post-FDAAA. Overall, 37 of 165 (22%) trials were not published, precluding primary effectiveness outcome comparison. Among 120 of 128 (94%) published trials, the primary endpoint specified in the FDA documents was the same endpoint specified as primary in the publication; among these, the primary endpoint result reported in the FDA documents was the same or within 5% of the result reported in the publication for 120 (100%) trials. Rates of concordant primary effectiveness outcome reporting were not significantly different between published pre-FDAAA and post-FDAAA trials (24 of 28 (86%) vs 96 of 100 (96%); *p* = 0.07) (Table 5).

**Table 5:** Outcome reporting and interpretation concordance of published clinical trials supporting US FDA cardiovascular device approvals between 2005 and 2020, pre- and post-FDAAA (*n* = 128)

	Number of published studies (%)	Concordant outcome reporting, *n* (%)	Risk ratio (*95% CI*)	Concordant interpretation, *n* (%)	Risk ratio (*95% CI*)
Overall	128 (100)	120 (94)		120 (94)	
Trial completion date			1.12 (0.96 to 1.31)		0.96 (0.88 to 1.06)
Pre-FDAAA	28 (22)	24 (86)		27 (96)	
Post-FDAAA	100 (78)	96 (96)		93 (93)	
*p* value			0.07		0.44

*CI* confidence interval, *FDAAA* FDA Amendment Act

**Fig. 2:**
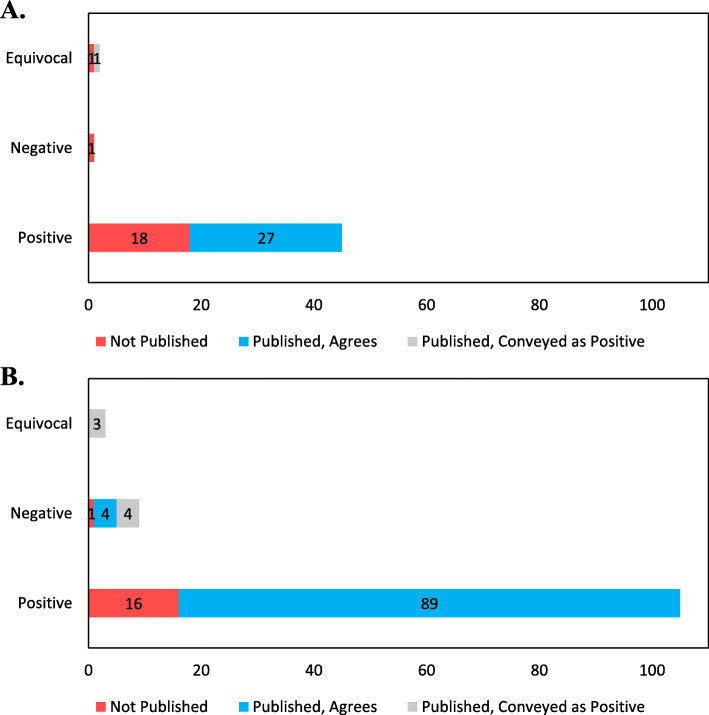
US FDA reviewer trial interpretation and publication, along with a published interpretation of the trial findings, for novel cardiovascular devices approved by the US FDA between 2005 and 2020, pre- and post-FDAAA. US FDA reviewer trial interpretation as positive, equivocal, or negative. **A** Pre-FDAAA; **B** Post-FDAAA

Among the 48 pre-FDAAA trials, FDA reviewers characterized 45 (94%) as positive, 2 (4%) as equivocal, and 1 (2%) as negative, whereas among the 117 post-FDAAA trials, FDA reviewers characterized 105 (90%) as positive, 3 (3%) as equivocal, and 9 (8%) as negative (Fig. 2). Overall, 116 of 150 (77%) positive trials were published, 4 of 5 (80%) equivocal trials, and 8 of 10 (80%) negative trials. Among published trials, rates of concordant interpretation were not significantly different between pre-FDAAA trials and post-FDAAA trials (27 of 28 (96%) vs 93 of 100 (93%); *p* = 0.44). All 8 discordant trials, 1 pre-FDAAA and 7 post-FDAAA, were published in a manner that conveyed a more positive interpretation than that of the FDA reviewer. No trials were published in a manner that conveyed a more negative interpretation than that of the FDA reviewer.

## Discussion

In our study of all pivotal studies supporting high-risk cardiovascular devices approved by the FDA through the PMA pathway from 2005 to 2020, we found that implementation of FDAAA, which mandated clinical trial registration and result reporting, was associated with higher rates of pivotal trial registration, result reporting, and publication, but no differences in the accuracy of outcome reporting and trial interpretation among published studies. These results suggest that the legislation has improved transparency and unbiased result reporting of clinical trials, potentially mitigating selective publication and outcome reporting, which can thereby ensure that patient care decisions are based on more complete and accurate research.

Our study demonstrates that 98% of post-FDAAA trials were registered on ClinicalTrials.gov, 85% reported their results on ClinicalTrials.gov, and 85% were published in the peer-reviewed literature. These rates are high but lag behind those reported for new drugs [9, 13]. Impressive clinical trial registration and result reporting after FDAAA enactment was expected given the explicit requirement to require trial registration among all trials investigating FDA-regulated products. However, there is still room for improvement because 2% of post-FDAAA trials remain unregistered on ClinicalTrials.gov, 15% have not posted their results on ClinicalTrials.gov, and 15% remain unpublished in the peer-reviewed literature. As required by law, rates of registration and reporting of results on ClinicalTrials.gov should be 100%. Potential reasons for the reported inability to reach 100% may include publications reporting on different cohorts than the original trials or that trials were published before observations accrued or after statistical analyses were refined [24]. Continued study, building on prior investigations examining characteristics of trials associated with lower rates of registration and reporting of results on ClinicalTrials.gov, and trial publication, is warranted. Additionally, follow-up studies will be needed to see whether these rates persist or improve.

Among post-FDAAA trials, publication rate in the peer-reviewed literature was higher when compared to the rate of 80% observed among studies supporting FDA approval of novel, high-risk cardiovascular devices between January 2011 and December 2013 [25]. Although it continues to greatly exceed the rate of 49% observed for trials supporting FDA-approved, high-risk cardiovascular devices between January 2000 and December 2010 [6], given that these pivotal trials represent the best evidence of medical device safety and effectiveness, there is no reason that the clinical and research community should not expect a publication rate of 100%. Also, 96% of published studies reported primary effectiveness outcomes in a manner concordant with FDA reviews, which is nearly identical to a prior study of medical device publication and result reporting [25]. Our rate may be more representative of concordant result reporting post-FDAAA because we analyzed 15 years of FDA approvals post-FDAAA while the aforementioned study analyzed 3 years post-FDAAA. Our specificity in matching the five elements for a defined endpoint (i.e., domain, measure, metric, method of aggregation, and timepoint) when determining primary effectiveness endpoint concordance may have reduced the rates that we report [22]. Future studies exploring the implications of primary endpoints switched within the FDA documents and between the FDA documents and the publications are warranted. Nonetheless, our results showed higher rates than the initial study of selective reporting for medical devices that reported a rate of 69% for both identical and similar primary endpoint reporting, which when binned most closely matched our “concordant outcome reporting” statistic, in pivotal high-risk cardiovascular device trials between 2000 and 2010 [6].

We found that 93% of published trial interpretations were concordant with FDA reviews, which is slightly lower than a prior study of medical device publication and result reporting [25] but again may be more representative of concordant interpretation reporting post-FDAAA because of our larger sample size. There were no significant differences in rates of publication when examining device and design characteristics among pre- and post-FDAAA trials, which differs from a previous report of cardiovascular device research that found both publicly held company sponsors and life-sustaining device designation to be associated with the likelihood of publication [25]. Of note, the FDAAA Final Rule, which clarifies and expands the regulatory requirements and procedures for submitting registration and result information for certain trials to ClinicalTrials.gov, became effective on January 18, 2017, and responsible parties were required to comply on April 18, 2017 [26, 27]. In our analysis, 23 studies were completed after implementation and 20 studies were completed after responsible parties were required to comply. Despite FDAAA not being fully implemented until 2017 with The Final Rule, we nevertheless observed a large associated impact. Due to the recency of The Final Rule, future investigation on the impact of this policy is warranted.

There are several limitations to be considered in the interpretation of our findings. First, we only looked at trials supporting FDA approval of high-risk cardiovascular devices. Our results may not be generalizable to all high-risk medical devices and should be confirmed for FDA approvals in other therapeutic areas. Second, our study is cross-sectional and observational, and we can only establish associations, not causal conclusions, about the impact of FDAAA. Third, we limited our search of trial registration to ClinicalTrials.gov and excluded the use of other clinical trial registration sites. That said, US law requires trial registration of FDA-regulated products on ClinicalTrials.gov. Fourth, our study was focused on the reporting and publication of primary efficacy endpoints and interpretations and did not examine reporting or publication of secondary efficacy and safety endpoints. Finally, publication interpretations depended on if they acknowledged a success or failure in meeting their target effectiveness endpoints. Standardized methods for this decision would have allowed for greater reproducibility and potentially more accuracy [28].

## Conclusion

FDAAA was associated with higher rates of clinical trial registration on ClinicalTrials.gov, result reporting, and publication in the peer-reviewed literature for trials supporting FDA approval of high-risk cardiovascular medical devices. Among published trials, rates of accurate primary efficacy outcome reporting and trial interpretation were high and no different post-FDAAA. These findings have important implications for understanding the potential impact of the FDAAA and informing future policy and regulatory efforts to ensure transparency and unbiased results reporting of the clinical trials supporting FDA approval of high-risk medical devices.

## Data Availability

The datasets used and/or analyzed during the current study are available from the corresponding author on reasonable request.

## Abbreviations

FDA: Food and Drug Administration
FDAAA: Food and Drug Administration Amendment Act
PMA: Premarket Approval
AED: Automated External Defibrillator
NCT: National Clinical Trial
STROBE: Strengthening the Reporting of Observational Studies in Epidemiology
